# Medical Education

**Published:** 1855-10

**Authors:** 


*Medical Education.*—We are glad to see in our exchanges an increasing disposition to discuss this important topic. That the system of Medical Education commonly adopted in this country is very incomplete; or more properly defective in many important particulars, is evident to all who have devoted proper attention to the subject. Those who anticipated a rapid removal of all these defects, through the instrumentality of the American Medical Association, have been disappointed, and a few of them ready to characterize the Association itself a failure on this account. The latter, however, but poorly appreciated, either the obstacles to be overcome or the time required for their removal. To point out effects is generally an easy task.

To devise and execute appropriate remedies, especially where the interests of many are concerned, is a task far more difficult and perplexing. It is a common remark that all real reforms or advances which affect important interests move slowly; and the history of Medical Education in this country certainly does not militate against the truth of the maxim. Yet there is progress. And the influence of the National organization is being felt throughout the whole country. The absence of the chairmen of the committees on Medical Education from the two last meetings of the Association, and the consequent absence of any report on the subject, caused those meetings to pass by with less discussion than on former occasions.

But members did not, on that account feel less interest in the general subject. The most important action taken at the last meeting, in reference to it, was the appointment of a special committee to report at the next meeting, on the following resolutions offered by Dr. A. Stille, of Philadelphia, viz.:

"*Resolved*, That a special committee of five be appointed to report at the next meeting of the Association on the following question: Might not the present system of repeating the same Lectures to the same classes, during two successive terms, be usefully modified by extending the Lectures of each chair over two sessions, so as to embrace a systematic and complete discussion of each of the following subjects:

1st. Special Regional, and General Anatomy, including illustrative references to Morbid Anatomy.

2d. Inorganic, Organic, Pharmaceutical Chemistry, and Toxicology.

3d. General and Human Physiology, Hygiene, Medical Jurisprudence.

4th. Medical Botany, Materia Medica, General Therapeutics.

5th. General Pathology, Morbid Anatomy, (systematic) Practice of Medicine.

6th. General Surgical Pathology or Institutes of Surgery, Mechanical, Operative, and Medical Surgery.

7th Obstetrics, Diseases of Women, and Diseases of Children.

8th. Hospital Clinical Medicine and Surgery.

*Resolved*, That the committee, at *an early day*, address the several Medical Colleges, in regard to the proposed plan of instruction, requesting from them an official expression of opinion upon its merits and feasibility."

The following gentlemen constitute the committee as appointed by the President of the Association, viz. : Drs. A. Stilli, Samuel Jackson, and John Bell, of Philadelphia, John Watson, of New York, and J. L. Cabell, of Va.

These are names well known to the profession, and we shall expect a most able and interesting report on the subject at the next meeting.

The leading idea embraced in the proposition, is, that the Lecture term in our colleges is too short to permit the several professors to present a full and complete course of Lectures on all the topics that should be embraced in their several departments. But instead of lengthening each term, it is proposed to make each course of Lectures extend over two consecutive college sessions. The idea here advanced is undoubtedly correct. A college term of four or five months is certainly too short to present a full course of instruction on all the important branches of medical science. But the remedy proposed is liable to very serious if not fatal objections. In the first place, the extension of each course of Lectures over two successive annual college terms, would render it necessary that the same class of students should always attend the same college two successive years, and that new students could only be consistently admitted at the commencement of each alternate year or college term. This, to say the least, would be a very great inconvenience. But another and even stronger objection is, that the proposed remedy or change, does not in the least obviate one of the most serious evils connected with our present system of medical instruction, namely, the crowding upon the mind of the student a large number of lectures each day, and simultaneously on topics the most elementary and the most complex and practical. Students who have studied medicine only a few weeks and those who have studied two and three years are required to listen to the same lectures; and the professors of practical medicine and surgery, are aften lecturing on the details of practice in their respective departments before those of Physiology, Anatomy, and Materia Medica have passed over the first elementary principles of our science. Dr. Stilli's proposition which seems to contemplate the giving of the same number of lectures by the same number of professors each term, only extending the course of each so as to reach through two terms, does not remedy in any degree this greatest of all the defects in our present system of medical college instruction. Simply lengthening the college term, or lengthening each course of lectures so as to embrace two terms, will not meet the wants of the profession or remedy the defects of the present system. But if we double the length of the present annual college session, and then divide it into *two terms*; so that the elementary branches can be taught or lectured on during one of the terms, and the practical branches during the other; then students in the early part of their studies could attend the term during which lectures were given on branches appropriate to the period of their study only the first year, and the term suited to their further progress the next year. By such an extension and division of the annual college session, the number of chairs could be increased, the whole field of medical science more perfectly included; while a smaller number of lectures would be given daily, and what is more then all, the student could receive his instruction on the several branches in their natural order or succession, instead of having them all crowded upon the mind heterogeneously. If all our medical colleges would confer together and adopt such a plan, and the profession at large throughout its social organization also adopt some systematic plan for ensuring the attainment of a proper preliminary education, on the part of all young men, before they are permitted to enroll themselves as students of Medicine with any respectable practitioner; it would soon result in a vast improvement in the education and usefulness of the whole profession in this country.

